# Draft genome sequence and resistome analysis of multidrug-resistant *Klebsiella pneumoniae* ST147 strain FM2_IFST_BCSIR with high mobility potential from hospital wastewater in Dhaka, Bangladesh

**DOI:** 10.1128/mra.00306-26

**Published:** 2026-07-02

**Authors:** Md. Rubiath Islam, Isthiaque Ahmed, Rajib Sarkar, Subarna Sandhani Dey, Mohammad Rubel Hossen Patwary, Monzur Morshed Ahmed, Abhijit Chowdhury

**Affiliations:** 1 Food Microbiology Research Laboratory, Institute of Food Science and Technology, Bangladesh Council of Scientific and Industrial Research 614904, Dhaka, Bangladesh; 2 Department of Biochemistry and Molecular Biology, Shahjalal University of Science and Technology 113074, Sylhet, Bangladesh; 3 Central Analytical and Research Facilities, Bangladesh Council of Scientific and Industrial Research 130051, Dhaka, Bangladesh; Wellesley College, Wellesley, Massachusetts, USA

**Keywords:** multidrug resistance, draft genome sequence, environmental microbiology

## Abstract

We report the draft genome sequence of *Klebsiella pneumoniae* FM2_IFST_BCSIR, a multidrug-resistant ST147 strain recovered from hospital wastewater in Dhaka, Bangladesh. The genome harbored 23 putative antimicrobial resistance genes across 6 antibiotic classes, including *blaNDM-5* and *blaOXA-48*, with integron and plasmid-associated genes indicating high dissemination and mobility potential.

## ANNOUNCEMENT

Carbapenemase-producing *Klebsiella pneumoniae* is associated with treatment failure, and its environmental release may promote dissemination of resistance genes into the community (1, 2). To our knowledge, whole-genome sequencing of high-risk *Klebsiella pneumoniae* from Bangladeshi hospital wastewater (HWW) has not been reported. Here, we present the molecular characterization of a multidrug-resistant (MDR) *K. pneumoniae* strain FM2_IFST_BCSIR isolated from Bangladeshi HWW.

HWW samples were collected in July 2025 from drains of a tertiary care hospital (23.7110^o^N, 90.4010^o^E) in Dhaka. Samples were serially diluted, and 100 µL from each dilution was spread-plated onto MacConkey Agar (HiMedia) and incubated at 37°C for 24 h. Large mucoid pink colonies were presumptively identified as *K. pneumoniae* and confirmed by Gram-staining, indole, methyl red and Voges-Proskauer test, urease, and citrate tests (3). Antibiotic susceptibility testing was performed using disk diffusion method with clinically important antibiotics, following Clinical and Laboratory Standards Institute guidelines (3, 4).

Bacterial DNA was extracted from pure culture grown overnight at 37°C in Luria Bertani Broth (HiMedia) using the DNeasy Blood and Tissue Kit (Qiagen) following the manufacturer’s protocol. Libraries were prepared with the Illumina DNA Prep (M) Tagmentation kit (Illumina). Sequencing was performed on an Illumina NextSeq-2000 generating 2 × 2,568,095 paired-end 150-bp reads with 111× genome coverage. Read quality was assessed with FastQC_v0.11.9, and trimming was performed using fastp_v0.20.1. Filtered reads were assembled *de novo* with SPAdes_v3.14.1 using—isolate parameter, and assembly quality was evaluated using QUAST_v5.2.0 and CheckM2_v1.1 (5, 6). Taxonomic assignment was performed using Kraken2 with standardDB (7). Species identity, multilocus sequence typing (MLST), virulence loci, capsule type, and O-antigen were determined using Kleborate_v2.2.0 and Kaptive_v2.0.3 (8). Contigs were annotated with Prokka_v1.14.6 (9). Resistance genes were identified using ResFinder_v4.1 (10). PlasmidFinder_v2.0 and PathogenFinder_v1.1 were used from the Center for Genomic Epidemiology (http://genomicepidemiology.org/). Integrons were detected using IntegronFinder_v2.0.6 (11). All tools were run with default parameters unless otherwise stated.

**Fig 1:**
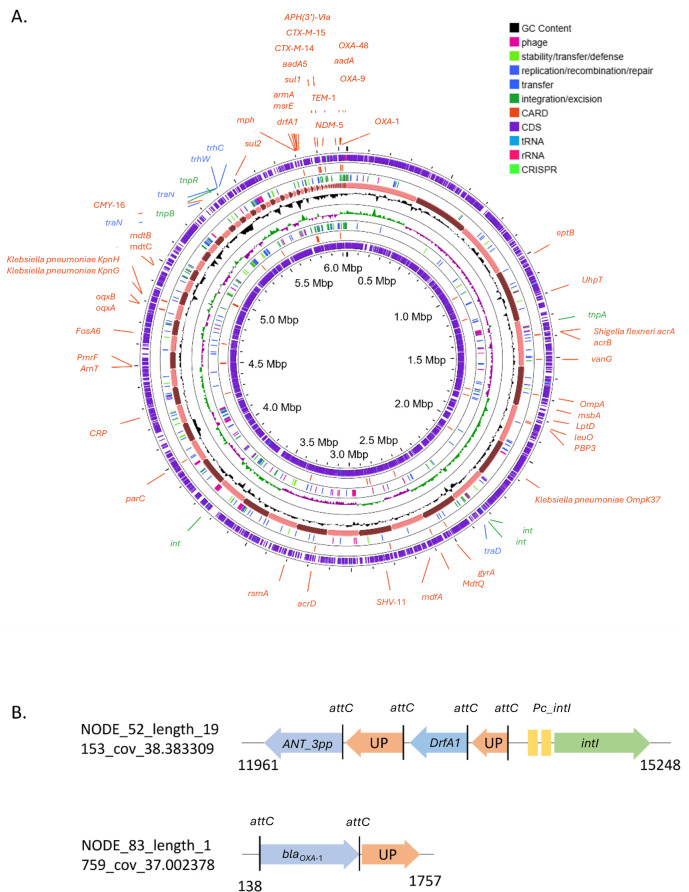
(**A**) Visualization of the draft genome assembly of *Klebsiella pneumoniae* FM2_IFST_BCSIR generated using the Proksee server (https://proksee.ca/), showing annotated features, antimicrobial resistance genes, and mobile genetic elements. This visualization is based on a multi-contig draft assembly. (**B**) Gene neighborhood map of the predicted integron cassette region containing putative resistance-associated genes. Class 1 integron integrase (intI1), aminoglycoside resistance (ant(3″)-Ia), trimethoprim resistance (dfrA1), β-lactam resistance (blaOXA-1), unknown-function protein (UP), recombination site (attC), and integron promoter (Pc_intI1).

FM2_IFST_BCSIR was classified as *K. pneumoniae* phylogroup Kp1, ST147, and serotype K64:O2α. The draft genome assembly was 6,038,492 bp in length, comprising 99 contigs with an N50 of 173,436 bp, an L50 of 13, and a GC content of 56.44% with CheckM2-estimated completeness and contamination of 100% and 1.91%, respectively. Prokka annotation predicted 5,735 protein-coding genes, 4 ribosomal RNAs, and 64 transfer RNAs. The strain showed MDR phenotype, showing resistance to aztreonam, cefotaxime, ciprofloxacin, gentamicin, trimethoprim-sulfamethoxazole, amoxicillin-clavulanic acid, and meropenem but susceptible to polymyxin B. Resistome analysis identified putative ARGs conferring resistance to β-lactams (*blaNDM-5*, *blaOXA-48*, and *blaCTX-M-15*), fluoroquinolones (*qnrS1* and *oqxAB*), aminoglycosides (*armA*), trimethoprim/sulfonamides (*sul1* and *sul2*), fosfomycin, and macrolides (Fig. 1A). Mutations in *gyrA* (Ser83Ile), *parC* (Ser80Ile), *ompK36* (134_135insGlyAsp), and *ompK35* (Arg60fs) were identified. The genome contained a complete class 1–like integron and two CALIN regions, and multiple plasmid replicons, including *IncC*, *IncHI1B*, *IncFIB*, *IncR*, and *ColRNAI* (Fig. 1B). The strain was predicted to be human pathogenic (0.809 probability score) and contained virulence genes, including *iuc1*, *AbST63*, and *ybt9/YbST584-2LV*.

## Data Availability

The sequence is available in National Center for Biotechnology Information database under BioProject PRJNA1399457 and BioSample SAMN56383707. The raw reads are available under Sequence Read Archive accession SRR37524480, and the assembly is available under accession JBXAYO000000000.
